# Expression of Cytokines, Chmokines and Growth Factors in Patients Undergoing Cataract Surgery with Femtosecond Laser Pretreatment

**DOI:** 10.1371/journal.pone.0137227

**Published:** 2015-09-02

**Authors:** Hui Chen, Haotian Lin, Danying Zheng, Yuhua Liu, Weirong Chen, Yizhi Liu

**Affiliations:** State Key Laboratory of Ophthalmology, Zhongshan Ophthalmic Center, Sun Yat-sen University, Guangzhou, Guangdong, China; Boston University School of Medicine, UNITED STATES

## Abstract

**Purpose:**

To describe cytokines, chemokines and growth factors profiles in patients undergoing cataract surgery with femtosecond laser pretreatment and investigate their relationships with the postoperative in vivo inflammation index.

**Methods:**

Aqueous humor was collected from 22 eyes after femtosecond laser pretreatment and from 22 eyes at the beginning of routine cataract surgery. The levels of 45 inflammation-related mediators were measured using multiplex fluorescent bead-based immunoassays. Laser flare photometry was measured preoperatively and at 1 day, 7 days and 30 days postoperatively.

**Results:**

Compared with the control group, the femtosecond laser treatment group showed significantly higher aqueous humor levels of fibroblast growth factor (FGF-2), tumor necrosis factor (TNF)-α, leukemia inhibitor factor (LIF), interleukin (IL)-1ra and IL-18, and significantly lower aqueous humor levels of IL-9, platelet-derived growth factor (PDGF)-BB, eotaxin and TNF-β. Postoperative aqueous flare was significantly greater in the manual cataract surgery group at 1 day (p<0.001), 7days (p<0.001) and 30 days (p = 0.002).No correlation was found between the analyzed mediators and the aqueous flare values.

**Conclusions:**

The expression profiles of cytokines, chemokines and growth factors and the correlations of these profiles with the in vivo inflammatory indexes for patients undergoing cataract surgery with femtosecond laser pretreatment were identified. Our data indicate a disturbance of postoperative inflammation response after femtosecond laser treatment.

## Introduction

In recent years, femtosecond laser technology has been introduced to the field of cataract surgery and offers many potential benefits. It has been reported that the femtosecond laser produces accurate self-sealing corneal incisions, allows for precise anterior capsulotomy, and reduces ultrasound energy during phacoemulsification.[1–4]

However, postoperative inflammation and the intraocular effect caused by surgical trauma and laser treatment are relatively unknown. It is not known whether the lens fragmentation of femtosecond laser-assisted cataract surgery, which leads to an increase in particulate matter in the anterior chamber, may exacerbates inflammation.[5,6] Previous studies have showed that postoperative aqueous flare was significantly greater with the manual cataract surgery group than with laser surgery.[7,8] Schultz et al reported that prostaglandins increase immediately after femtosecond laser treatment.[9] Postoperative inflammation is associated with the breakdown of the blood-aqueous barrier and is indicated by surgical trauma-induced cytokine production. Previous studies have described alterations of the cytokine levels in aqueous humor after various laser treatments. For example, increased levels of interleukin 1-beta (IL-1β), IL-6, IL-8, and tumor necrosis factor-alpha (TNF-α) have been reported in rabbit vitreous humor after retinal laser photocoagulation and rabbit aqueous humor after argon and neodymium:yag laser iridectomy.[10,11] To date, the profile of aqueous cytokines in patients after femtosecond laser treatment has not been described. Therefore, exploring a greater number of inflammation-relative mediators would provide broader insight into the inflammatory mechanisms involved in femtosecond laser pretreatment.

Recently, the multiplex bead immunoassay has been used to detect cytokines in tears, vitreous humor and the aqueous humor because its capacity to simultaneously quantify multiple cytokines in small sample volumes.[12] In this study, we used the multiplex bead immunoassay to evaluate the profiles of 45 cytokines, chemokines and growth factors in the aqueous humor after femtosecond laser pretreatment.

## Patients and Methods

### Patients

This was a prospective consecutive investigator-masked nonrandomized parallel cohort study performed at a single center. This study was performed in accordance with the Declaration of Helsinki and with the approval of the Sun Yat-sen University-Zhongshan Ophthalmic Center-Institutional Review Board (SYSU-ZOC IRB). Written informed consent was obtained from all patients. Consecutive patients with senile cataract who were scheduled to undergo femtosecond laser–assisted cataract surgery or manual cataract surgery with insertion of an intraocular lens (IOL) were enrolled in the study. All patients were given the option to undergo femtosecond laser pretreatment. Patients electing to undergo femtosecond laser–assisted cataract surgery were placed in the laser group. Based on our preliminary data, at least 22 patients needed to be included in the analysis to achieve sufficient power in the statistical calculations. The 22 cases in the study group were paired 1:1 with patients from a database of 50 consecutive cases who underwent conventional cataract surgery according to age, sex and PNS. A previous study showed that the average photon per millisecond for normal eyes is 4.24 (SD 1.12), with a range of 1.8–8.2.[13] Therefore, patients were excluded from the study if they had preoperative flare of more than 10 (ph/ms), a history of inflammatory or infectious eye diseases, previous ocular surgery or trauma, glaucoma, exfoliation syndrome, diabetic retinopathy, age-related macular degeneration, or used topical or systemic anti-inflammatory or anti-infectious agents.

Only one eye, which fulfilled all inclusion criteria and none of the exclusion criteria, was designated as the study eye in each patient; in patients in whom both eyes fulfilled all inclusion criteria and none of the exclusion criteria one randomly chosen eye was included for the purpose of statistical analysis.

### Measurement of aqueous flare

Aqueous flare was measured with a laser flare meter (FC-2000, Kowa, Tokyo, Japan) using methods that had been described previously.[14] Measurements were taken within 1 week before surgery and 1, 7 and 30 days after surgery. Flare values were measured under scotopic conditions without pharmacologic pupil dilation. Two different examiners obtained five measurements from the studied eye, and the results were averaged after excluding all measurements affected by artifacts.

### Surgical technique

All surgeries were performed at the Zhongshan Ophthalmic Center (Guangzhou, China). All manual and femtosecond laser surgeries were performed by the same experienced surgeon (Yi L). Prior to surgery, all patients were treated with topical ofloxacin four times daily for 3 days. [15] According to our standard protocol, no nonsteroidal anti-inflammatory drugs were administered before surgery.

In the femto group, cataract surgery was performed using the LenSx femtosecond laser (Alcon Laboratories, Fort Worth, TX, USA). Corneal applanation was performed using the SoftFit interface (Alcon Laboratories, Fort Worth, TX, USA), and the individual patient treatment programmed into the laser. Anterior capsulotomy (size 5.0 mm, 6 μJ pulse energy), lens-fragmentation(10μJ pulse energy), primary and side-port corneal incisions (6 μJ pulse energy) and corneal relaxing incisions (if required) were then created with the femtosecond laser under Optical Coherence Tomography (OCT) image control. A standardized lens-fragmentation pattern (3 cross-sections with a chop diameter of 5.2 mm and 1 central chop cylinder with a diameter of 3.0 mm) was used.

In the control group, standard cataract surgery was performed on all patients as described previously. [16] In both groups, following hydrodissection, the surgery was completed with standard phacoemulsification using the Infiniti Vision System Unit (Alcon Inc., Fort Worth, TX, USA), automated irrigation/aspiration to remove the cortex. A single-piece AcrySof SN60WF IOL (Alcon, Inc., Fort Worth, TX, USA) was implanted in the capsular bag.

### Aqueous humor sampling

In the femto group, within 5 minutes after laser treatment, the primary incision was opened under sterile conditions, and undiluted aqueous humor samples (0.1–0.2 ml) were aspirated into a syringe. The samples were immediately frozen and stored at −80°C until analysis. Similar to the femto group, a limbal paracentesis was performed with a sterile tuberculin syringe at the beginning of cataract surgery before making the initial incision; aqueous humor was collected from the paracentesis site. Both groups of samples were collected between July 2014 and August 2014 and stored for a similar interval under the same conditions.

### Multiplex analysis of cytokines in the aqueous humor

The concentrations of 45 human aqueous humor mediators were measure using a MAGPIX instrument (Luminex Corporation, Texas, USA), the xPONENT software (version 4.2.1324.0) and a ProcartaPlex Human Cytokine/Chemokine/Growth Factor Panel (eBioscience San Diego, CA, USA). The analysis procedure was conducted according to the manufacturer’s instructions:

1. cytokines: interleukin (IL)-1ra, IL-1β, IL-1α, IL-2, IL-4, IL-5, IL-6, IL-7, IL-9, IL-10, IL-12 p70, IL-13, IL-15, IL-17A, IL-18, IL-21, IL-22, IL-23, IL-27, IL-31, tumor necrosis factor(TNF)-α, TNFβ/LTA, interferon-γ (IFN-γ), IFN-α, brain derived neurotrophic factor (BDNF), granulocyte-macrophage colony stimulating factor (GM-CSF), leukemia inhibitor factor (LIF) and stem cell factor(SCF);
2. chemokines: IL-8/CXCL8, eotaxin (CCL11), growth regulated oncogene (GROα)/CXCL1, interferon inducibleprotein-10 (IP-10)/CXCL10, monocyte chemoattractant protein (MCP)-1/CCL2, macrophage inflammatory protein-1α(MIP-1α)/CCL3, MIP-1β/CCL4, regulated upon activation, normal T cell expressed and presumably secreted(RANTES)/CCL5 and stromal cell derived factor 1α (SDF1α)/CXCL12;
3. growth factors: epidermal growth factor (EGF), fibroblast growth factor (FGF-2), hepatocyte growth factor (HGF), β-Nerve Growth Factor (β-NGF), platelet derived growth factor (PDGF)-BB, placental growth factor (PIGF), vascular endothelial growth factor (VEGF)-A and VEGF-D.

### Statistical analysis

Data were recorded as the mean ± standard deviation (SD) or as the medians and 25th–75th interquartile range. The statistical analyses were performed using SPSS for Windows Version 17.0 (Chicago, IL,USA). The Pearson χ^2^ test was used to compare the proportions of the qualitative variables Multivariate analysis of covariance (MANCOVA) was used to compare quantitative data. The covariates evaluated for their impact on biomarker levels included sex, age, PNS (Pentacam nucleus staging) and aqueous flare before surgery and 1, 7 and 30 days after surgery. Comparisons of biomarker concentrations that were not normally distributed were followed by Box–Cox transformations to make the data distribution more normal. For cytokines that still did not meet the prerequisites for inclusion in the analysis after Box–Cox transformation, a weighted least-squares regression was conducted. The false discovery rate (FDR) method was used to perform a correction for multiple testing. [17] Spearman correlation coefficients were used to assess the correlation. A p value less than 0.05 and a q value less than 0.05 were considered statistically significant.

## Results

### Patient demographics

A total of 44 subjects (44 eyes) who fulfilled the inclusion criteria were enrolled in the study: 22 underwent femtosecond laser surgery and 22 were underwent conventional cataract surgery.

Patient demographics and clinical features are summarized in Table 1. The mean ages of the laser group and the cataract control individuals were 65.3 and 66.9 years, respectively (p = 0.642). There was no difference between the patient groups in their age, sex, or cataract density. Preoperative and postoperative courses were uneventful in all patients.

**Table 1:** Demographic and clinical features of all patients who underwent either femtosecond laser-assisted cataract surgery (Laser group) or manual cataract surgery (Control group).

Variables	Laser group	Control group	p
**Eyes**	22	22	
**age**	65.27±11.45	66.86±11.09	0.642
**sex(male/female)**	10/12	7/15	0.537
**Cataract staging (PNS mean)**	2.59±0.73	3.00±0.69	0.064
**Suction time(second)**	102.5±23.4		
**Laser time(second)**	45.5±5.4		
**Aqueous flare**			
**preoperative**	6.00±2.91	6.32±2.12	0.676
**postoperative**			
** 1 day**	15.05±3.82	22.67±4.60	**<0.001**
** 7 day**	9.80±3.91	14.81±3.33	**<0.001**
** 30 day**	6.00±2.91	6.32±2.12	0.676

PNS = Pentacam Nucleus staging. Bold values represent significance.

The difference in preoperative aqueous flare was not statistically significant between the groups. However, postoperative aqueous flare was significantly greater in the manual cataract surgery group than in the femto group at 1 day (p<0.001), 7 days (p<0.001) and 30 days (p = 0.002) after surgery.

### Cytokine/chemokine/growth Factor concentrations in aqueous humor

The concentrations of the assayed cytokines, chemokines and growth factors are summarized in Table 2. Compared with the control group, the concentrations of FGF-2 (p = 0.007,q = 0.031), IL-1ra (p = 0.015,q = 0.046), IL-18 (p<0.001,q = 0.027), LIF(p = 0.013,q = 0.042), and TNF-α (p = 0.006,q = 0.031) in the femtosecond laser treatment patients were significantly higher. However, the IL-9 (p = 0.008,q = 0.031), TNF-β (p = 0.004,q = 0.031), eotaxin (p = 0.007,q = 0.031) and PDGF-BB (p = 0.003,q = 0.030) concentrations were significantly lower in the femto group than in the control group. There were no significant differences in the concentrations of the other mediators’ concentrations between the femto and control group.

**Table 2:** Aqueous humor levels of Cytokines, Chemokines and Growth factor.

		Normally distributed	Laser group		Manual group		Statistics	P	Q
			Mean	Median	Mean	Median			
**Cytokines**	**IL-1ra**	No	214.21±201.73	122.86(43.23–354.18)	65.45±34.74	68.76(43.23–94.45)	6.510^△^ †	**0.015**	**0.046**
	**IL-1β**	Yes	0.94±0.70	0.87(0.37–1.40)	1.23±0.62	1.22(0.69–1.49)	0.310^△^	0.582	0.477
	**IL-1α**	Yes	1.14±0.38	1.15(0.88–1.45)	1.31±0.29	1.37(1.12–1.49)	0.170^△^	0.680	0.516
	**IL-2**	No	11.824±7.80	10.26(3.68–16.78)	12.78±9.04	10.26(3.68–16.78)	0.670^△^ †	0.417	0.400
	**IL-4**	Yes	8.85±2.52	8.53(6.69–10.44)	10.34±2.26	10.44(9.60–11.76)	0.080^△^	0.785	0.551
	**IL-5**	Yes	1.46±0.73	1.50(0.91–1.87)	1.76±0.85	1.87(1.13–2.24)	0.220^△^	0.644	0.502
	**IL-6**	No	101.11±124.10	47.16(17.41–166.89)	19.48±13.77	14.99(13.99–21.41)	3.230^△^ †	0.081	0.159
	**IL-7**	No	4.02±1.40	3.96(3.33–5.18)	3.80±1.20	3.54(3.18–4.88)	2.320^△^ †	0.137	0.209
	**IL-9**	Yes	90.57±3.85	87.08(71.48–100.07)	141.97±36.54	146.78(129.84–160.84)	7.900^△^	**0.008**	**0.031**
	**IL-10**	No	0.22±.41	0.08(0.03–0.21)	0.15±0.14	0.09(0.04–0.27)	0.030^△^ †	0.861	0.574
	**IL-12p70**	No	0.20±0.18	0.19(0.05–0.30)	0.32±0.24	0.29(0.10–0.43)	2.430^△^ †	0.127	0.202
	**IL-13**	Yes	6.00±2.48	6.36(3.59–7.95)	5.91±1.38	6.36(5.50–6.57)	0.160^△^	0.691	0.520
	**IL-15**	Yes	13.98±4.86	12.32(9.29–17.98)	14.57±4.65	15.09(13.32–17.67)	0.630^△^	0.433	0.407
	**IL-17A**	No	0.77±0.34	0.84(0.54–0.91)	0.89±0.36	0.84(0.54–0.91)	0.390^△^ †	0.537	0.457
	**IL-18**	No	1.15±1.06	1.23(0.04–1.76)	0.69±0.66	0.47(0.22–1.11)	5.227*	**<0.001**	**0.027**
	**IL-21**	Yes	9.78±4.59	9.34(6.48–11.90)	13.70±6.58	14.75(9.34–18.21)	0.500^△^	0.483	0.431
	**IL-22**	No	217.45±134.00	217.45(114.45–287.81)	182.22±87.32	208.06(125.15–248.29)	2.860^△^ †	0.099	0.178
	**IL-23**	Yes	55.41±19.65	55.49(41.39–65.73)	49.46±13.52	51.07(42.64–55.74)	0.840^△^	0.366	0.376
	**IL-27**	Yes	31.55±15.13	29.89(23.66–39.91)	34.24±9.53	34.89(28.64–39.91)	0.210^△^	0.648	0.504
	**IL-31**	No	46.84±15.44	51.58(40.30–57.16)	54.79±15.89	58.49(50.78–63.52)	0.880^△^ †	0.355	0.370
	**TNFα**	No	3.46±1.78	3.19(2.64–3.86)	3.11±0.96	2.64(2.64–3.73)	3.525*	**0.006**	**0.031**
	**TNFβ/LTA**	Yes	17.56±9.54	13.87(11.70–20.82)	33.16±9.13	34.14(26.92–40.79)	9.280^△^	**0.004**	**0.031**
	**IFNγ**	No	0.10±0.21	0.01(0.00–0.06)	0.09±0.15	0.01(0.00–0.14)	2.038*	0.058	0.130
	**IFNα**	No	0.75±0.50	0.62(0.44–0.92)	0.97±0.21	1.00(0.83–1.11)	0.700^△^ †	0.408	0.396
	**GM-CSF**	Yes	21.02±7.05	21.45(14.01–25.17)	22.18±9.11	20.15(19.43–26.27)	0.010^△^	0.913	
	**BDNF**	Yes	2.17±1.12	2.02(1.43–2.83)	2.58±0.82	2.62(2.08–3.06)	0.220^△^	0.643	0.502
	**LIF**	No	6.96±6.87	4.21(2.55–8.80)	2.51±1.46	2.35(1.24–2.92)	2.620*	**0.013**	**0.042**
	**SCF**	Yes	4.32±1.45	4.81(3.21–5.51)	3.94±1.32	4.07(2.50–4.81)	3.880^△^	0.057	0.128
**Chemokines**	**Eotaxin(CCL11)**	No	3.52±2.31	3.59(2.15–5.04)	3.74±1.81	3.64(2.33–4.78)	8.430^△^ †	**0.007**	**0.031**
	**GROα(CXCL1)**	No	24.72±20.76	17.94(15.73–22.03)	16.32±2.22	17.05(15.25–17.70)	3.460^△^ †	0.071	0.147
	**IP-10(CXCL10)**	Yes	26.07±15.04	25.41(16.28–36.75)	11.42±12.29	7.41(3.27–13.52)	1.540^△^	0.222	0.285
	**MCP-1(CCL2)**	Yes	587.75±338.07	550.08(379.13–636.99)	569.90±185.22	538.39(474.14–706.11)	0.580^△^	0.452	0.414
	**MIP-1α(CCL3)**	Yes	3.80±2.34	3.29(2.71–4.45)	3.87±1.42	3.67(2.92–4.48)	0.070^△^	0.799	0.556
	**MIP-1β(CCL4)**	No	23.46±9.82	24.42(18.28–29.49)	26.34±4.52	26.48(23.99–29.27)	-1.424*	0.164	0.235
	**RANTES(CCL5)**	Yes	1.27±0.63	1.22(0.88–1.70)	1.59±0.49	1.69(1.56–1.85)	1.270^△^	0.267	0.318
	**SDF1α(CXCL12)**	No	211.97±47.98	209.28(186.83–232.66)	217.88±37.94	208.45(194.14–241.25)	-0.797*	0.431	0.406
	**IL-8(CXCL8)**	No	26.40±18.30	19.61(16.73–27.52)	25.50±6.33	26.98(23.56–28.93)	0.185*	0.854	0.572
**Growth Factors**	**EGF**	No	1.73±1.24	1.74(1.10–2.07)	2.18±0.87	2.28(1.63–2.94)	2.570^△^ †	0.118	0.195
	**FGF-2(FGFbasic)**	No	608.31±249.21	560.36(487.83–751.13)	23.41±7.54	23.00(18.05–28.15)	2.895*	**0.007**	**0.031**
	**HGF**	No	730.22±332.58	676.95(497.26–998.28)	362.67±331.71	284.06(129.47–451.41)	3.480^△^ †	0.070	0.146
	**βNGF**	Yes	23.40±11.56	23.64(14.01–30.87)	21.92±7.43	23.00(17.06–27.47)	0.360^△^	0.553	0.464
	**PDGF-BB**	No	9.89±6.57	8.59(2.47–14.77)	13.13±10.08	13.95(7.06–18.48)	10.070^△^ †	**0.003**	**0.030**
	**PIGF**	Yes	10.16±2.90	10.08(8.41–12.34)	11.64±1.95	11.87(10.17–13.12)	0.170^△^	0.680	0.506
	**VEGF-A**	No	193.00±123.72	183.25(144.15–236.98)	238.31±155.44	214.30(143.98–303.50)	1.830^△^ †	0.185	0.254
	**VEGF-D**	No	2.57±2.08	2.02(0.90–4.20)	3.59±1.67	2.80(2.41–5.30)	1.130^△^ †	0.295	0.336

Data are expressed as the mean±SD (pg/ml) and median, 25th–75th interquartile range (pg/ml). Bold values represent significance.

IL = interleukin; TNF = tumor necrosis factor; IFN = interferon; GM-CSF = granulocyte-macrophage colony stimulating factor; BDNF = brain derived neurotrophic factor

LIF = leukaemia inhibitor factor; SCF = stem cell factor; CCL = C-C motif ligand; CXCL = C-X-C motif ligand; GROα: growth regulated oncogene; IP = interferon inducibleprotein; MCP = monocyte chemoattractant protein macrophage; MIP = inflammatory protein-1αregulated upon activation; RANTES = normal T cell expressed and presumably secreted; SDF = stromal cell derived factor; EGF = epidermal growth factor; FGFbasic = fibroblast growth factor-basic; HGF = hepatocyte growth factor; bNGF = β-nerve growth factor

PDGF-BB = platelet derived growth factor BB chain; PIGF = lacental growth factor; VEGF = vascular endothelial growth factor

^△^ F value by Multivariate analysis of covariance (MANCOVA).Age, sex, PNS and preoperative, postoperative day 1, day 7 and day 30 aqueous flare were forced into the model

† Based on Box–Cox transformation.

* t value by weighted least squares regression

P = P-value, significance level for MANCOVA and weighted least squares regression (significance is P<0.05)

Q = Q-value, a correction for multiple testing (false discovery rate FDR,Q<0.05).

### Association between cytokine/chemokine/ growth factor concentrations and inflammatory variables

For both groups, the results showed that the aqueous flare value increased rapidly after surgery on day 1 and then gradually decreased during the first month after surgery. No correlation was found between the altered mediator levels and the postoperative day 1 flare value in the femto group (Table 3).

**Table 3:** Correlation between concentrations of the assayed mediators in aqueous humor and clinical characteristics of patients underwent femtosecond-laser cataract surgery.

	Suction time		Laser time		postoperative 1 day flare	
	R	p	r	p	r	p
**IL-9**	-0.133	0.555	0.329	0.135	-0.230	0.303
**IL-18**	-0.083	0.713	0.349	0.112	0.372	0.088
**TNF**α	-0.034	0.879	-0.016	0.943	0.155	0.490
**TNFβ**	-0.216	0.334	0.021	0.927	0.019	0.933
**IL-1RA**	-0.265	0.233	0.345	0.116	-0.012	0.958
**Eotaxin**	-0.303	0.171	0.100	0.658	-0.061	0.788
**LIF**	-0.100	0.659	-0.229	0.305	-0.373	0.087
**PDGF-BB**	-0.253	0.257	0.328	0.136	-0.083	0.713
**FGF-2**	-0.126	0.576	0.076	0.737	-0.042	0.851

IL = interleukin; TNF = tumor necrosis factor; LIF = leukaemia inhibitor factor;

PDGF-BB = platelet derived growth factor BB chain; FGF = fibroblast growth factor

### Correlation Analyses

We also evaluated whether the duration of the femtosecond laser procedure affected the mediator aqueous humor levels in the femto group. No significant correlation was found between suction time and the mediator concentrations in the femto group (p>0.05). Furthermore, no correlation between laser time and mediator concentrations was observed in this study (p>0.05).

### Complications

No eyes developed anterior capsule tears, posterior capsule rupture, zonular dehiscence, vitreous prolapse or loss, phaco burn, or phaco bite. There was no docking abort or suction loss during imaging or femtosecond laser treatment. No adverse events occurred during the 4-week follow-up period.

## Discussion

Femtosecond laser–assisted cataract surgery became available recently and its safety and efficacy have been established; however, little is known about the inflammatory response of eyes to laser pretreatment. The objective of our study was to investigate the profiles of released cytokines, chemokines and growth factors after femtosecond laser pretreatment and to assess the degree of postoperative inflammation.

In this study, we observed that the concentrations of FGF-2, LIF, IL-1ra, TNF-α and IL-18 were significantly higher in the femto group than in the control group. In addition, the aqueous humor levels of IL-9, eotaxin, PDGF-BB and TNF-β were significantly lower in the femto group than in the manual group. These results indicate a disturbed balance in the levels of cytokines, chemokines and growth factors in eyes after femtosecond laser treatment.

Among the 9 cytokines that exhibited significant differences in expression between the groups, FGF-2 LIF and TNFα have been shown to be associated with the development of posterior capsular opacification (PCO). It has been reported that increased levels of these cytokines and growth factors in aqueous humor in the early phase after cataract surgery could induce lens epithelial cells (LEC) proliferation, migration and transdifferentiation in the early phase.[18] FGF-2 is known to play an important role in proliferation, migration, and fiber differentiation of normal LECs. [19,20] Previous studies have shown that FGF-2 induces LEC proliferation, which may contribute to the development of PCO. [21] Similarly, TNF-α gene expression in LECs was found in lens capsule samples from cataract surgery and may induce postoperative inflammation and LEC proliferation.[22] LIF was also found to exert regenerative and proliferative responses and may also promote cell proliferation.[23] This result suggests that the increased levels of these mediators, which have been shown to induce proliferation, may generate a potential risk for the development of postoperative PCO. It is possible that immediately after surgery, the exposure of LECs to increased levels of FGF-2, TNF-α and LIF could eventually result in their proliferation and in the formation of PCO. However, a recent clinical study showed that femtosecond laser- assisted capsulotomy was a safe procedure relative to the postoperative PCO rate.[24] A possible explanation for this discrepancy is that only a slight decrease in PCO formation was observed in that study and that the follow-up time was only 18 months. Therefore, further studies with long-term follow-up are still needed to evaluate the clinical significance of these results.

Other cytokines and chemokines related to underlying inflammation and immune response, such as IL-1ra and IL-18, were also found to be significantly increased in aqueous humor samples from the femto group. IL-1ra is a natural anti-inflammatory cytokine that serves as a modulator of immune responses by regulating the agonist effects of IL-1. [25,26] IL-1ra has been reported to inhibit myofibroblast formation in a rat model of healing.[27] Therefore, the significantly higher expression of IL-1ra in the aqueous humors of patients with femtosecond laser pretreatment may increase the inhibition of IL-1 signaling and make the eyes less susceptible to inflammation related complications, such as capsular contraction syndrome. In contrast, IL-18, a pro-inflammatory cytokine, is a T lymphocyte chemokine with profound effects on neutrophils.[28] Recent evidence suggests that IL-18 may function as an anti-angiogenic agent in the eye.[29] Numerous studies have demonstrated the ability of IL-18 to prevent neovascularization in the retina, choroid and cornea.[30] A previous study also demonstrated a protective role for IL-18 in the development of wet AMD.[31] Because it has been reported that cataract surgery is associated with increased risks of the formation and progression of AMD, the increased level of IL-18 observed in the femto group may have a role in protecting the formation of AMD after cataract surgery. [32]

Interestingly, we also observed decreased level of IL-9, eotaxin, PEGF-BB and TNF-β in the aqueous humors of the femto group. IL-9 belongs to the γc family of cytokines, which induce the proliferation of activated T cells, epithelial cells and B cells and broadly contribute to lymphocyte proliferation, differentiation and survival.[33] Therefore, some anti-inflammatory responses to T cells may be lost with the down-regulation of this functional γc family cytokine. Of equal importance, other inflammatory cytokines, such as TNF-β, eotaxin and PDGF-BB, were also decreased in patients with femtosecond laser treatment. TNF-β is involved in the mediation of inflammatory reactions and endothelial function.[34] Eotaxin is an eosinophil-specific chemoattractant that has been found to be elevated in allergic conditions and other systemic inflammatory disorders. [35,36] Additionally, PDGF has been shown to stimulate cell division and is closely related to VEGF.[37] Based on our experimental results, we believe that the decreased levels of inflammation-related mediators indicate that a functional disturbance of the immune response associated with breakdown of the blood-aqueous barrier, uveal reaction and systemic response is provoked by femto laser pretreatment. Further studies are needed to determine the true role of these cytokines in patients treated with femto laser.

Consistent with previous reports, our study showed that patients who underwent femtosecond laser pretreatment experienced a rapid increase in aqueous flare value on day 1 after surgery and that this level then gradually decreased during the first month after surgery. No significant association was found between aqueous flare value and the increased concentrations of the assayed mediators in the femto group.

### Strength of the study

First, instead of collecting serum samples, we collected aqueous humor samples during cataract surgery, which likely provided a more accurate reflection of the intraocular inflammatory status. Second, aqueous humor cytokines were detected by multiplex bead immunoassay, which measures multiple cytokines simultaneously using a comparatively small sample volume. Finally, profiles of up to 45 cytokines were analyzed in this study, which provides a great amount of information for determining the mechanisms involved in the response of the eye to femtosecond laser pretreatment.

### Limitations of the Study

One limitation of this study was that topical corticosteroids and NSAIDs were used during the postoperative period. Although patients in both groups were given the same drugs and were asked to take them at the same frequency and for the same duration, confounding results cannot be ruled out because of potential differences in patient compliance between the groups.

## Conclusions

In conclusion, femtosecond laser pretreatment in cataract surgery significantly induces altered levels of profibrotic intraocular cytokines which are involved in the development of PCO. The increased levels of some anti-inflammatory and anti-angiogenic cytokines may exert a protective influence on certain inflammation-related complications. Decreased levels of immune-related cytokines also indicate a disturbance of postoperative inflammation response after laser treatment. It is unknown whether this is solely due to the surgical trauma or due to other processes. Future studies are needed to investigate the detailed role of the alter levels of mediators in postoperative ocular inflammation as a previously uninvestigated aspect of femtosecond laser cataract surgery.
